# Case Report: Malignant peripheral nerve sheath tumor of the kidney with a novel granular cell morphology

**DOI:** 10.3389/fonc.2025.1559861

**Published:** 2025-10-16

**Authors:** Danli Ye, Guangning Yan, Wenzhi Cui, Xuwen Lai, Wenyuan He, Chengyong Lei, Wei Wang

**Affiliations:** ^1^ Department of Pathology, General Hospital of Southern Theater Command, People’s Liberation Army of China, Guangzhou, China; ^2^ Department of Urology, Nanfang Hospital, Southern Medical University, Guangzhou, China; ^3^ Graduate School, Guangzhou University of Chinese Medicine, Guangzhou, China

**Keywords:** malignant peripheral nerve sheath tumor, kidney, granular cell, H3K27me3, case report

## Abstract

Malignant peripheral nerve sheath tumors (MPNSTs) arising from the kidney are rare. The present report describes a renal MPNST of a 33-year-old man exhibiting novel morphological features. Histologically, the tumor was comprised of spindle Schwann cells and granular-like tumor cells. The latter are characterized by large, round-to-polygonal cells with abundant, finely granular eosinophilic cytoplasm, which form variable nodules or are diffusely distributed among the spindle tumor elements. Immunohistochemistry revealed both tumor components expressed CD56, Leu-7, PGP9.5, and Nestin, indicating a neural crest origin. A complete loss of H3K27me3 expression confirmed the diagnosis of MPNST. The patient had no history of neurofibromatosis type 1. The granular cell changes in renal MPNST expand the known morphological spectrum of MPNSTs.

## Introduction

Malignant peripheral nerve sheath tumors (MPNSTs) are spindle cell neoplasms that typically arise from peripheral nerves, showing variable differentiation toward nerve sheath cellular components, such as Schwann cells, fibroblasts or perineurial cells. They can occur sporadically or in patients with neurofibromatosis type 1. MPNSTs occur most frequently in the extremities, particularly proximally, followed by the trunk, head and neck. Previous reported have documented lesions in parenchymal organs such as the cervix, skull, heart, lung, adrenal gland and retroperitoneum (1–6). However, MPNSTs originating from the kidney are particularly rare (7–9). Heterologous differentiation, including skeletal muscle, bone, cartilage, blood vessels and glandular differentiation, is observed in approximately 15% of MPNST tumors. To date, granular cell changes have not been reported. This study reports a renal MPNST with granular cell differentiation and review the current literature, hoping to further expand knowledge of MPNSTs.

## Case presentation

**Figure 1:**
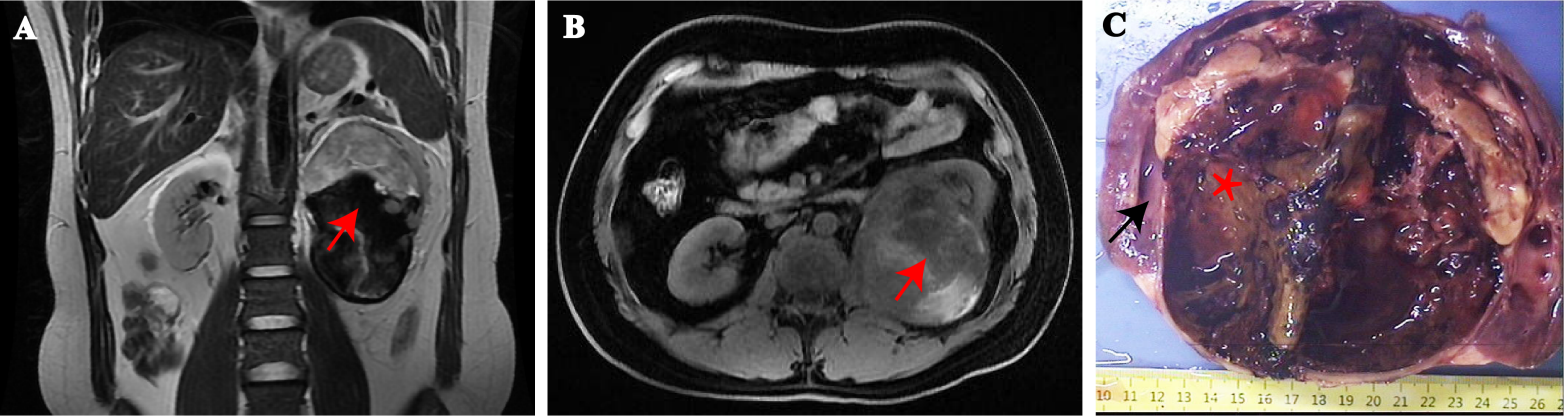
MRI showing a large tumor arising from the hilum of the left kidney. **(A)** Sagittal and **(B)** transverse view. (red arrows = tumor). Gross findings, the mass was located at the renal hilum and clearly demarcated from the surrounding renal tissue **(C)**. (black arrows = renal tissue, asterisk = tumor).

A 33-year-old overweight male patient (BMI, 25.9) presented with dull, constant left lumbar pain, accompanied by nausea, vomiting, and subsequent hematuria for 1 week. He had no family history of cancer or neurofibromatosis type 1. He did not smoke, drink, have irregular sleep habits, or frequent exposure to high temperatures and chemicals at work. Biochemical examination showed elevated low-density lipoprotein (LDL; 4.2 mmol/l). A large cystic-solid mass was found in the left kidney during a routine ultrasound examination. CT and MRI showed a large, encapsulated cystic-solid mass involving the left kidney, with gradual enhancement on post-contrast images, raising the possibility of renal cell carcinoma in differential diagnosis. The tumor measured ~13.4 × 9.8 × 7.8 cm, with relatively clear boundaries (**Figures 1A, B**). A left radical nephrectomy and lymph node dissection were performed.

**Figure 2:**
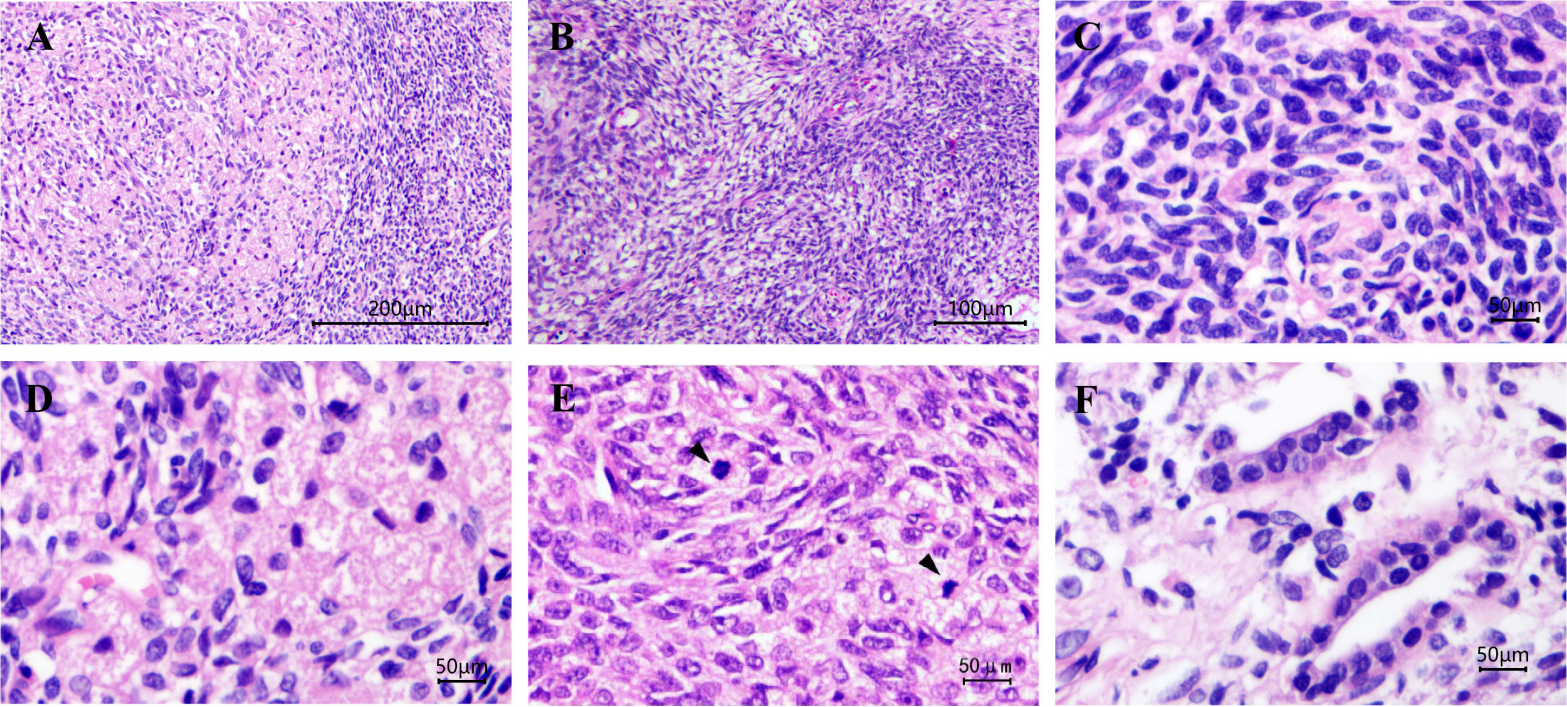
Malignant peripheral nerve sheath tumor with granular cell-like changes. The granular-like tumor cells either formed nodules of variable size or were diffusely distributed among the spindle cells **(A)** Magnification, x100. The tumor cells are arranged in a pattern of alternating density, presenting a marbling appearance **(B)** magnification, x200. **(C)** Spindle cells had elongated, buckled or wavy nuclei with hyperchromatic nuclei. **(D)** Granular cells were large, with granular and eosinophilic cytoplasm. **(E)** Mitoses were more easily found in granular-like tumor cells compared with the spindle elements and **(F)** normal renal tubules were frequently entrapped at the periphery. Magnification, x400. Black arrows indicate mitoses.

**Figure 3:**
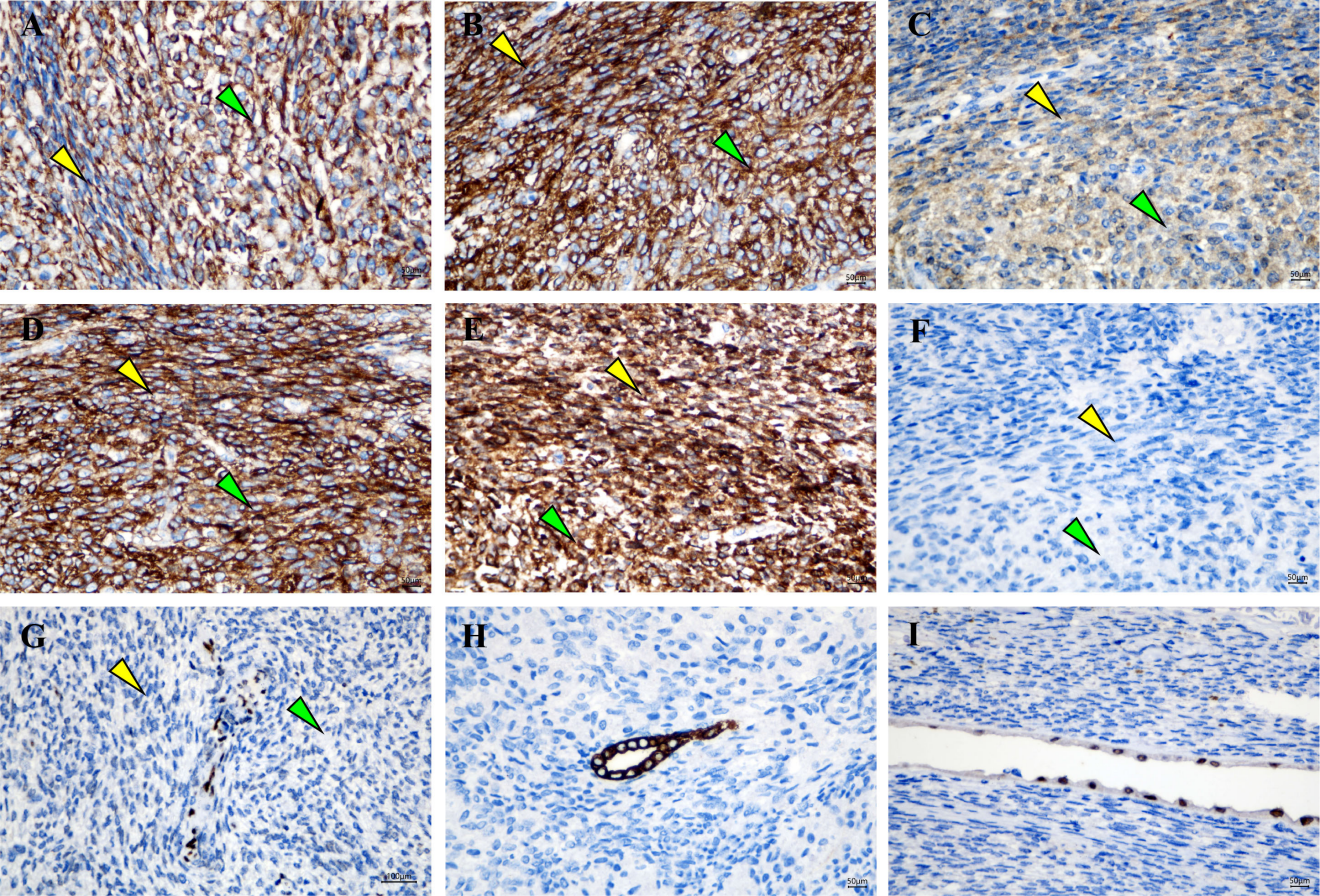
Granular-like tumor cells and spindle cells showed immunoreactivity for **(A)** Nestin, **(B)** Leu-7, **(C)** PGP9.5, **(D)** CD56 and **(E)** BCL2. **(F)** Negative staining for S‑100 protein. **(G)** A complete expression loss for H3K27me3 was shown in tumor cells, wheres the endothelial cells were nucleated positive. Entrapped renal tubules were positive for **(H)** keratin7 and **(I)** PAX8. Magnification, x400. Green arrows indicate Granular-like tumor cells and yellow arrows indicate spindle cells.

Grossly, A protruding, round mass was found in the kidney, measuring approximately 13.0×10.0×8.0 cm at the renal hilum, sharply demarcated from the surrounding renal parenchyma, with a distinct fibrous pseudo-capsule. The cut surface of the mass exhibited a grayish-red color, a soft consistency, and a fish-flesh-like appearance. Additionally, areas of gelatinous, solid and hemorrhage areas were observed (**Figure 1C**). Microscopically, the tumor was composed of spindle tumor cells reminiscent of Schwann cells and large granular-like cells with eosinophilic cytoplasm. The granular-like cells were either distributed modularly or diffusely interspersed with the spindle cells (**Figures 2A, B**). The spindle tumor cells had minimal amphophilic cytoplasm and showed elongated, buckled or wavy nuclei with hyperchromatic or vesicular nuclei (**Figure 2C**). The granular-like cells were round-to-polygonal, with distinct cell borders and abundant, finely granular eosinophilic cytoplasm (**Figure 2D**). Nuclei varied in morphology, ranging from uniformly small and mildly hyperchromatic to larger and vesicular with distinct nucleoli. Mitoses, including atypical mitoses, were more easily found in granular-like cells compared with the spindle elements (**Figure 2E**). The normal renal tubules were frequently entrapped at the periphery (**Figure 2F**). The gelatinous areas are attributed to the presence of myxoid stroma, necrosis and hemorrhagic components within the tumor. Immunohistochemistry showed that both the spindle and granular-like tumor cells were positive for Nestin, Leu-7, PGP9.5, CD56 and BCL2 (**Figures 3A–E**). Tumor cells were negative for S-100 (**Figure 3F**), SOX10, Desmin, MyoD1, GFAP, keratin, EMA, WT1, CD34, STAT6, HMB45, Melan-A, P16, P53 and MDM2. Both tumor components showed complete loss of H3K27me3 staining, with retained expression in endothelial cells (**Figure 3G**). The entrapped renal tubules show positive staining for keratin7 and PAX8 (**Figures 3H, I**). The renal cell carcinoma was excluded due to spindle-shaped tumor cells and immunohistochemistry showed negative expression of keratin and EMA. A diagnosis of high-grade MPNST was confirmed, which was further classified as T2N0MX, stage IIIA.

## Discussion

MPNSTs are aggressive soft-tissue sarcomas that are typically associated with NF1, while approximately 40% of MPNSTs arise sporadically after radiation exposure. Renal MPNSTs are particularly rare that only 11 cases were reported from 2001–2023 in PubMed (7–17). The present study reported a patient who had a large mass in the renal hilus. Microscopically, the perivascular accentuation of tumor cells, hemangiopericytoma-like pattern, bright and dark appearance, cells with both a blunt and pointed end support the diagnosis of MPNST. In certain areas of the tumor, large epithelial-to-polygonal tumor cells with intensely granular, eosinophilic cytoplasm were present. These cells developed light-stained nodules of varying sizes or were interspersed with spindle Schwann cells. Immunoreactivity for CD56, Leu-7, PGP9.5 and Nestin was observed in both tumor components, which suggested a neural crest origin. A complete loss of H3K27me3 confirmed the diagnosis of MPNST (18, 19). Other spindle cell sarcomas that often arise from the kidney, such as synovial sarcoma and nephroblastoma, should be differentially diagnosed from MPNSTs. Synovial sarcoma typically shows focal keratin or EMA expression with possible H3K27me3 loss. MPNSTs also lack the specific t(X;18) translocation that is specific to synovial sarcoma (20). The entrapped renal tubules can be misdiagnosed as the epithelial component of classical nephroblastoma. Nephroblastomas characteristically contain undifferentiated blastemal cells and typically show WT1 immunoreactivity which is absent in MPNSTs (21). In addition, the plump, polygonal tumor cells with intensely eosinophilic cytoplasm and visible mitotic figures closely resemble a malignant granular cell tumor (GCT), which can be distinguished by strong, diffuse S-100 and SOX10 expression in GCT and its rarity in deep soft tissue sites.

GCT can be derived from Schwann cells. The inactivation of ATPase H^+^ Transporting Accessory Protein 1 (ATP6AP1) or ATP6AP2 leads to decreased vacuolar-type ATPase activity, which results in the aggregation of particles in the cytoplasm of Schwann cells (22). It has been previously reported that ATP6AP1 can affect lipid metabolism in humans (23). The LDL cholesterol levels of the patient were significantly elevated in this patient. Whether the granular cell changes are related to abnormal lipid metabolism requires further investigation from clinicians into whether additional treatment is required.

There are few reports on the molecular mechanism of sporadic MPNST (24). Reports show recurrent mutations in PRC2 core components EED and SUZ12 in MPNST, leading to global loss of H3K27me3. The loss of H3K27me3 expression is a useful diagnostic tool for high-grade MPNST. Hirbe et al. reported the oncogenic *BRAF V600E* mutation. In addition, *TP53* mutations have also been identified in sporadic MPNSTs. Longo et al. showed that sporadic MPNSTs may also have the Cyclin Dependent Kinase Inhibitor 2A (CDKN2A)/p16 mutations, which are common in NF1-associated MPNSTs. *BRAF V600E* was detected using PCR and *CDKN2A*/p16 and *TP53* mutation was detected using immunohistochemistry. In this study, the results showed that *BRAF* gene and *CDKN2A*/p16 were not mutated, however *TP53* mutation was detected Other rare mutations, including DNA Methyltransferase 1, Nuclear Mitotic Apparatus Protein 1 and Neurotrophic Receptor Tyrosine Kinase 1 have been previously reported.

The present study novelly reported the granular cell changes of renal MPNST (25). Both the spindle and the granular-like tumor cells showed the same immunophenotype, but the latter had a higher mitotic rate. Divergent differentiation of MPNSTs into skeletal muscle/rhabdomyosarcoma, heterologous osteoid/osteosarcoma, chondroid/chondrosarcoma are common, but into angiosarcoma and epithelial elements are rare (26–29). Granular cell changes associated with monomorphic spindled cells in humerus MPNSTs were previously reported by Ortiz (30), Focal expression of SOX10 and the complete loss of H3K27me3 confirmed the diagnosis. Our findings expand the known morphological spectrum of renal MPNSTs.

## Conclusions

We reported a case of MPNST with a rare origin and unique morphological appearance Despite the interference of widely granular cell changes in malignant spindle sarcoma, typical morphological changes of MPNSTs and expression loss of H3K27me3 confirmed diagnosis. This special appearance further expands the morphological spectrum of MPNSTs and assists in making definite diagnosis.

## Data Availability

The original contributions presented in the study are included in the article/supplementary material. Further inquiries can be directed to the corresponding authors.
